# Modification of an aggressive model of Alport Syndrome reveals early differences in disease pathogenesis due to genetic background

**DOI:** 10.1038/s41598-019-56837-6

**Published:** 2019-12-31

**Authors:** Sara Falcone, Laura Wisby, Thomas Nicol, Andrew Blease, Becky Starbuck, Andrew Parker, Jeremy Sanderson, Steve D. M. Brown, Cheryl L. Scudamore, Charles D. Pusey, Frederick W. K. Tam, Paul K. Potter

**Affiliations:** 1 Mammalian Genetics Unit, Medical Research Council, Harwell science and innovation campus, Oxford, OX11 0RD UK; 2 Mary Lyon Centre, Medical Research Council, Harwell science and innovation campus, Oxford, OX11 0RD UK; 3 Renal and Vascular Inflammation Section, Department of Medicine, Imperial College, London, W12 0N UK; 4 Department of Biological and Medical Sciences, Faculty of Health and Life Sciences, Oxford Brookes University, Oxford, OX3 0BP UK

**Keywords:** Podocytes, Alport syndrome

## Abstract

The link between mutations in collagen genes and the development of Alport Syndrome has been clearly established and a number of animal models, including knock-out mouse lines, have been developed that mirror disease observed in patients. However, it is clear from both patients and animal models that the progression of disease can vary greatly and can be modified genetically. We have identified a point mutation in *Col4a4* in mice where disease is modified by strain background, providing further evidence of the genetic modification of disease symptoms. Our results indicate that C57BL/6J is a protective background and postpones end stage renal failure from 7 weeks, as seen on a C3H background, to several months. We have identified early differences in disease progression, including expression of podocyte-specific genes and podocyte morphology. In C57BL/6J mice podocyte effacement is delayed, prolonging normal renal function. The slower disease progression has allowed us to begin dissecting the pathogenesis of murine Alport Syndrome in detail. We find that there is evidence of differential gene expression during disease on the two genetic backgrounds, and that disease diverges by 4 weeks of age. We also show that an inflammatory response with increasing MCP-1 and KIM-1 levels precedes loss of renal function.

## Introduction

Mutations in one of the three collagen chains present in the adult glomerular basement membrane, COL4A3, A4 and A5^1,2^ can result in Alport Syndome (AS), a progressive renal disease ultimately resulting in end stage renal failure. Several animal models of AS have been developed and have led to a better understanding of the pathogenesis of this disease^3–5^. However, the lack of a comprehensive understanding of pathogenesis can be associated with there are no curative treatments outside dialysis and transplantation. In patient populations^3^ there is a wide spectrum of disease severity, and from animal models there is strong evidence for genetic modification of the pathways leading to renal failure^6,7^. The increased expression of *Col4a6* on the C57BL/6J background^7^ ameliorates murine AS but does not account for all of the strain specific effect on disease, and other autosomal loci are thought to contribute to a slower disease progression on this genetic background^6^. However, the mechanisms leading to strain specific differences in disease progression are unknown. Studies of animal models have also highlighted several pathways that can alter the progression of AS, including modulation of the immune response^8–10^. Modulation of the renin-angiotensin system with ACE-inhibitors has also been shown to ameliorate disease suggesting blood pressure is an important factor in disease^11^.

Alport Syndrome results in an altered composition of the glomerular basement membrane and ultimately results in podocyte effacement and loss of glomerular function^12,13^. A key readout of renal function is proteinuria and the presence of increased levels of urinary albumin can have a direct effect on podocytes^14,15^, so it is conceivable that GBM dysfunction could result in proteinuria which affects podocyte function resulting in a positive feedback loop eventually leading to ESRF^14^. Supporting this notion is the observation that proteinuria precedes podocyte effacement in another model of GBM dysfunction, Pierson Syndrome, where alterations in podocyte structure and reduced expression of slit diaphragm proteins occur subsequent to proteinuria^16^. Furthermore, deletion of albumin in a model of AS ameliorates disease^17^. However, high levels of proteinuria do not necessarily result in podocyte effacement^18–20^. The podocyte can contribute to deterioration of renal function by the secretion of monocyte chemoattractant protein-1 (MCP-1), matrix metallopeptidase 12 (MMP-12) and tumour necrosis factor (TNF-α)^14^ in response to stress.

Whilst the primary defect in AS is an alteration in the composition of the GBM, there is evidence that the tubular response is critical to the pathogenesis of AS^21–23^ and may indeed precede the deterioration of glomerular function. Signalling between the tubule and glomerulus can influence the response of the podocytes to a collagen deficiency in the GBM and ameliorate disease^9^.

We have identified a novel mutation of *Col4a4* resulting in a model of Alport Syndrome and have carried out a detailed analysis of disease progression and gene expression. Disease progression is greatly modified depending on genetic background, demonstrating C57BL/6J as a protective background^6,7,24^ and confirming a significant effect of an autosomal modifier. Examination of renal function, gene expression, and histopathology point to a divergence in disease progression after proteinuria and GBM alterations have occurred. A slower inflammatory response, less tubular damage, and prolonged podocyte health are the key phenotypes associated with the improved outcome on the C57BL/6J background.

The majority of AS models developed so far have had an aggressive course with disease proceeding to ESRF within a matter of a few weeks. Whilst such models have contributed significantly to our understanding of AS, the rapid disease progression can obscure the sequence of events during pathogenesis, thus preventing a detailed dissection of the processes occurring in the kidney. In addition to this, the more extreme disease present on these backgrounds provides a compressed time frame and in comparison, to what is generally seen in patients. The relatively slow progressive disease present on the C57BL/6J background provides a greater window of opportunity for studying mechanisms and trialling therapeutic interventions, and indeed may better model the progression of AS symptoms in patients.

## Results

### Identification of a Col4a4 mutant

**Figure 1:**
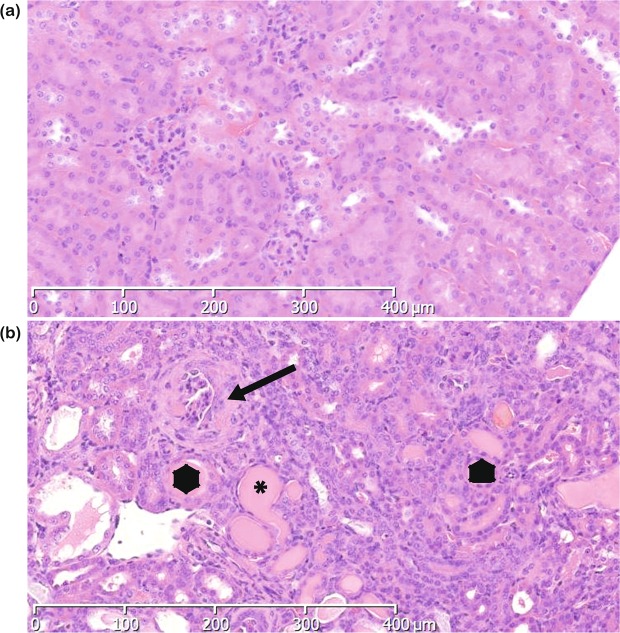
Histolopathology of mutant and wild type kidneys. Representative examples of kidneys H&E stained sections from **(a)** Col4a4^+/+^ and **(b)** Col4a4^G400X/G400X^ G3 mice at 53 days of age at x10 magnification are shown. Glomerulonephropathy was seen in Col4a4^G400X/G400X^ G3 mice and was characterised by diffuse involvement of glomeruli and tubules with enlargement, increased cellularity and sclerosis of glomeruli (black arrow) and degenerative and regenerative changes in tubules (tubular dilatation), hyaline casts (*).

**Figure 2:**
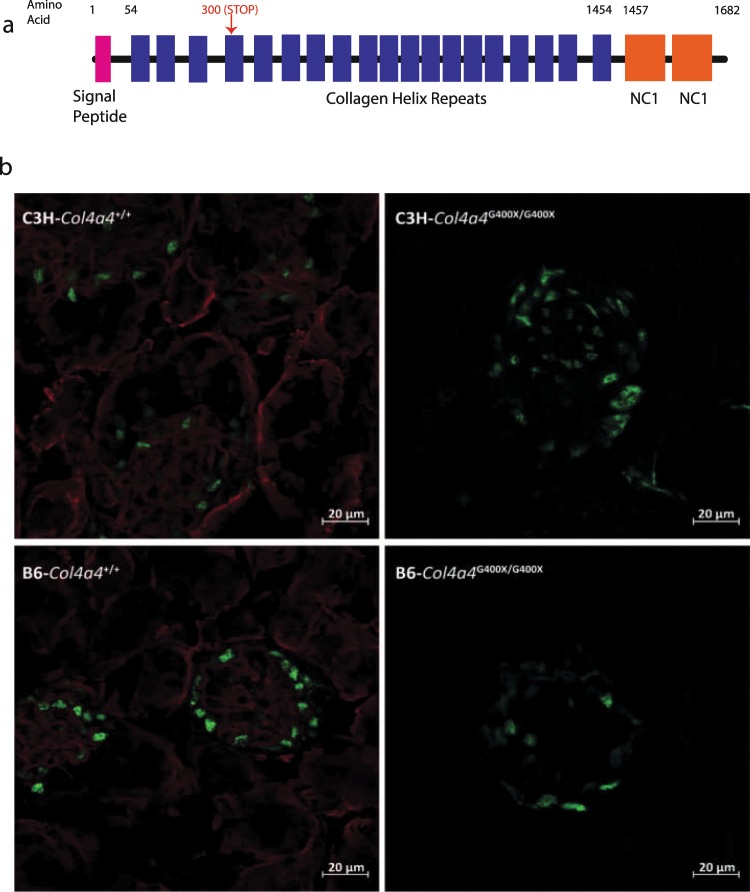
An early stop mutation in Col4a4 results in the absence of protein. **(a)** A diagrammatic representation of the position of the Col4a4^G400X^ mutation. **(b)** Immunofluorescence of representative wild type and mutant kidneys showing an absence of COL4A4 protein in mutant glomeruli. WT1 = green COL4A4 = red.

As part of ongoing phenotype driven screening programme^25^, mutant mice exhibiting end stage renal disease (ESRD) between 37 and 103 days of age were identified. Kidneys from affected mice were small and pale and histolopathological analysis of these mice revealed extensive glomerulonephopathy (Fig. 1 and Fig. S1). This was associated with increased levels of serum urea and creatinine (Table S1). The chromosomal region containing the causative mutation was mapped to between 80 and 83 Mb on chromosome 1, located in the type 4 collagen gene *Col4a4* and identified through whole genome sequencing as a mutation in a glycine codon leading to a stop codon at amino acid 400 (Fig. 2a). *Col4a4*^*G400X/G400X*^ mice were backcrossed for one generation onto C3H.Pde6b+^26^, then inter-crossed, and subsequent breeding studies confirmed this as the causative mutation with a complete absence of COL4A4 protein in mutant mice (Fig. 2b).

**Figure 3:**
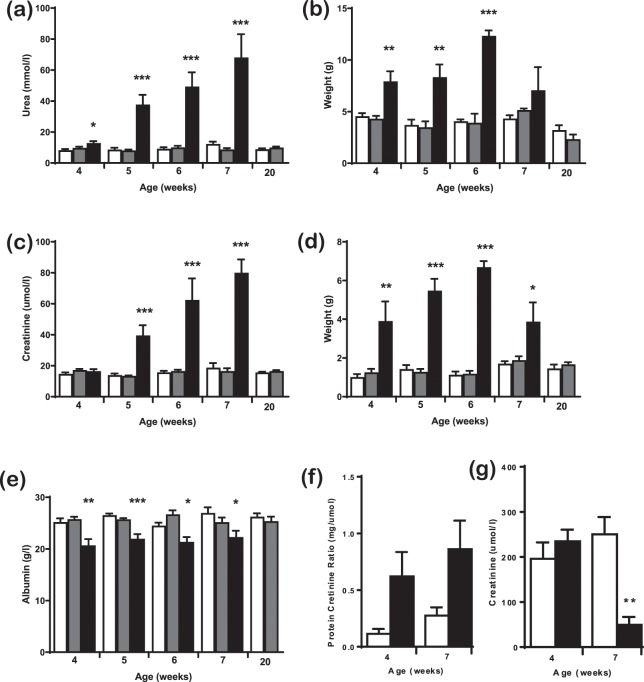
A time course of disease progression in wild type, heterozygous, and homozygous Col4a4^G400X^ mice. Mice were housed in metabolic caging for 24-hour urine collection and water consumption then terminally bled and plasma levels of various parameters determined by clinical chemistry analysis on an Olympus AU400 analyser. **(a)** Plasma urea **(b)** Plasma creatinine **(c)** Plasma albumin **(d)** Water intake **(e)** Urinary output **(f)** Total urinary protein:creatinine ratio and **(g)** creatinine clearance. Males and females were included in the analysis. White = wild type, Grey = heterozygous and Black = homozygous mice. For wild-type, heterozygous, homozygous respectively at; 4 weeks = 4, 22, and 7; 5 weeks = 8, 12, and 11; 6 weeks = 9, 9, and 4; 7 weeks = 5, 9, and 6; 20 weeks = 10 wild-type and 8 heterozygotes Data shown are mean ± S.E.M. and significance determined within each time point group using a one-way ANOVA with Tukey’s multiple comparisons test comparing, one way ANOVA with Tukey’s multiple comparison test *P < 0.05, **P < 0.01, ***P < 0.001 or a T-test for protein:creatinine ratio and GFR.

Mutations in *COL4A4* have been associated with Alport Syndrome^27^ and another ENU mutation in *Col4a4* has been identified which resulted in a slowly progressive disease^28^. However, our line appeared to have an aggressive, early onset phenotype. We carried out a time course on cohorts of *Col4a4*^*G400X/G400X*^, *Col4a4*^*G400X*/+^ and wild type mice. *Col4a4*^*G400X/G400X*^ mice were significantly lighter by 5 weeks of age, consumed more water and produced more urine from 4 weeks of age (Fig. 3a,b). Levels of albumin in the plasma of *Col4a4*^*G400X/G400X*^ mice were significantly lower than *Col4a4*^*G400X*/+^ and *Col4a4*^+/+^ mice at all of the time points analysed (Fig. 3c). Plasma levels of urea and creatinine increased (Fig. 3d,e), and urine levels decreased with time (Fig. 3b). Significant changes were observed as early as 4 weeks in homozygous mice. The protein:creatinine ratio was elevated in homozygous mice from 4 weeks of age, but not significantly, and the estimated glomeruar creatinine clearance decreased over time and was significantly lower in homozygous mice by 7 weeks of age (Fig. 3f,g). Histolopathological analysis revealed moderate glomerulonephropathy in the kidneys of some 4-week-old Col4a4^G400X/G400X^ mice with almost all animals having a marked diffuse glomerulonephropathy affecting the whole cortex from week 7 (Fig. 1). Few homozygous mice reached ten weeks of age before they were culled on welfare grounds (Fig. S1).

Plasma and urine biochemistry of 20-week-old *Col4a4*^*G400X*/+^ and *Col4a4*^+/+^ mice were also analysed, and there were no significant differences in any of the parameters between the two genotypes (Fig. 3). Indeed, heterozygous mice up to 2 years of age exhibited no loss of renal function (data not shown).

At 6–7 weeks of age *Col4a4*^*G400X/G400X*^ mice underwent visual and auditory phenotyping to investigate whether these mice developed ocular defects and deafness as is often seen Alport patients. Optokinetic drum scores^29^, slit lamp, and opthalmoscopic observations were not significantly different from wild type controls, and auditory brainstem response (ABR) thresholds were also similar to wild type controls (Fig. S2), in agreement with previous observations in a *Col4a4* mutant line^30^.

### Genetic background modifies disease progression

**Figure 4:**
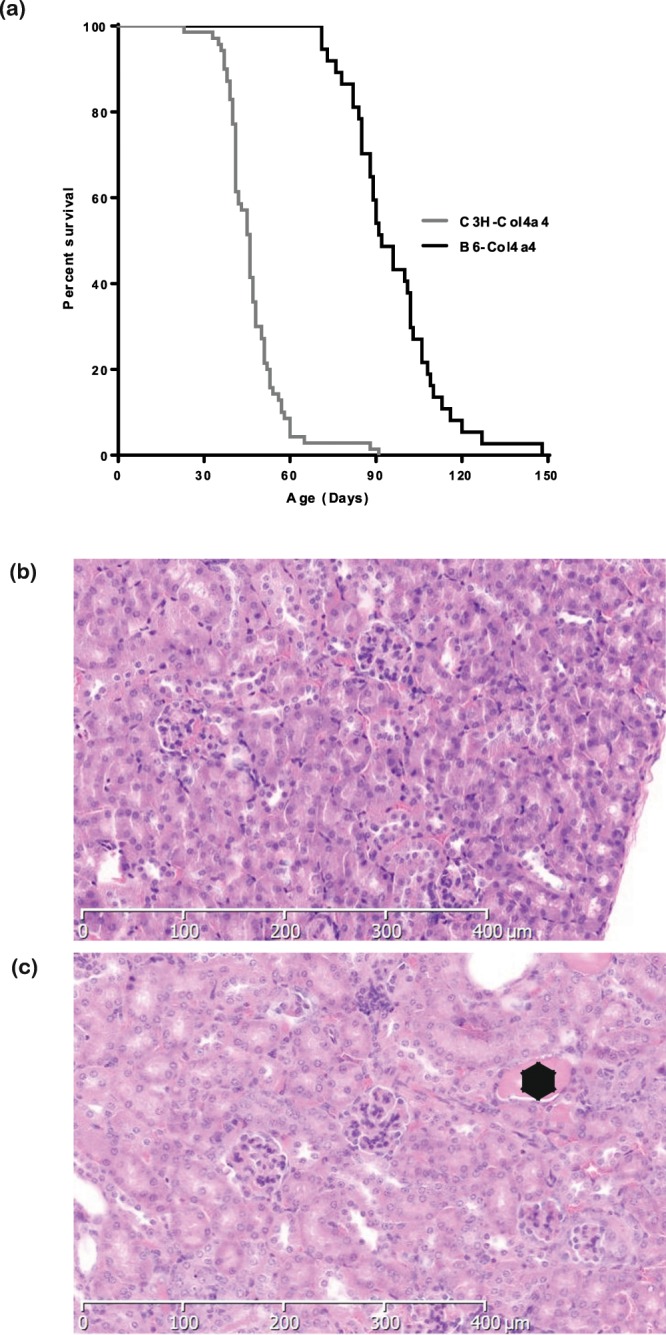
Genetic background has a significant effect on disease progression in Col4a4^G400X/G400X^ mice. **(a)** A Kaplan-Meyer plot showning is the age of homozygous Col4a4^G400X/G400X^ mice at which they reached welfare end points, thus indicating the mice were approaching ESRF. Data is from B6-Col4a4^G400X/G400X^ or C3H-Col4a4^G400X/G400X^, which refer to a single outcross of G3 mice to either C57BL/6J or C3H/HeH mice (C3H-Col4a4 n = 70, B6-Col4a4 n = 37). The log rank (Mantel-Cox) Test demonstrates a significant difference between the survival of B6-Col4a4^G400X/G400X^ or C3H-Col4a4^G400X/G400X^ mice (Chi square = 88.91, p < 0.0001) Histopathology of **(b)** B6-Col4a4^+/+^ and **(c)** B6-Col4a4^G400X/G400X^. Female mice 49 days of age are shown at x10 magnification. Glomerulonephropathy was not observed in B6-Col4a4^G400X/G400X^ mice, as was observed in C3H-Col4a4^G400X/G400X^ mice of this age (Fig. 1) but hyaline casts (*) and mild tubular basophilia were observed.

Mice from the original PEDV133 pedigree were on a mixed background of C3H.Pde6b+^26^ and C57BL/6J mice (approximately 63% C3H.Pde6b+). *Col4a4*^*G400X/G400X*^ PEDV133 mice have a mean survival time of 70 days, ranging from 37–103 days, suggesting an effect of the mixed genetic background on disease progression. To investigate the contribution of genetic background to the variation in disease we outcrossed *Col4a4*^*G400X/G400X*^ one generation to each strain and then, after inter-crossing, examined homozygotes. Outcrossing to C3H.Pde6b+ reduced the survival time to a mean of 47 days whereas on the C57BL/6J background the mean survival time was increased to 97 days. This resulted in a significant difference in the Kaplan Meyer curves depending on genetic background (Fig. 4a): C3H.Pde6b+ resulting in an earlier onset of disease and C57BL/6J appearing to be protective.

**Figure 5:**
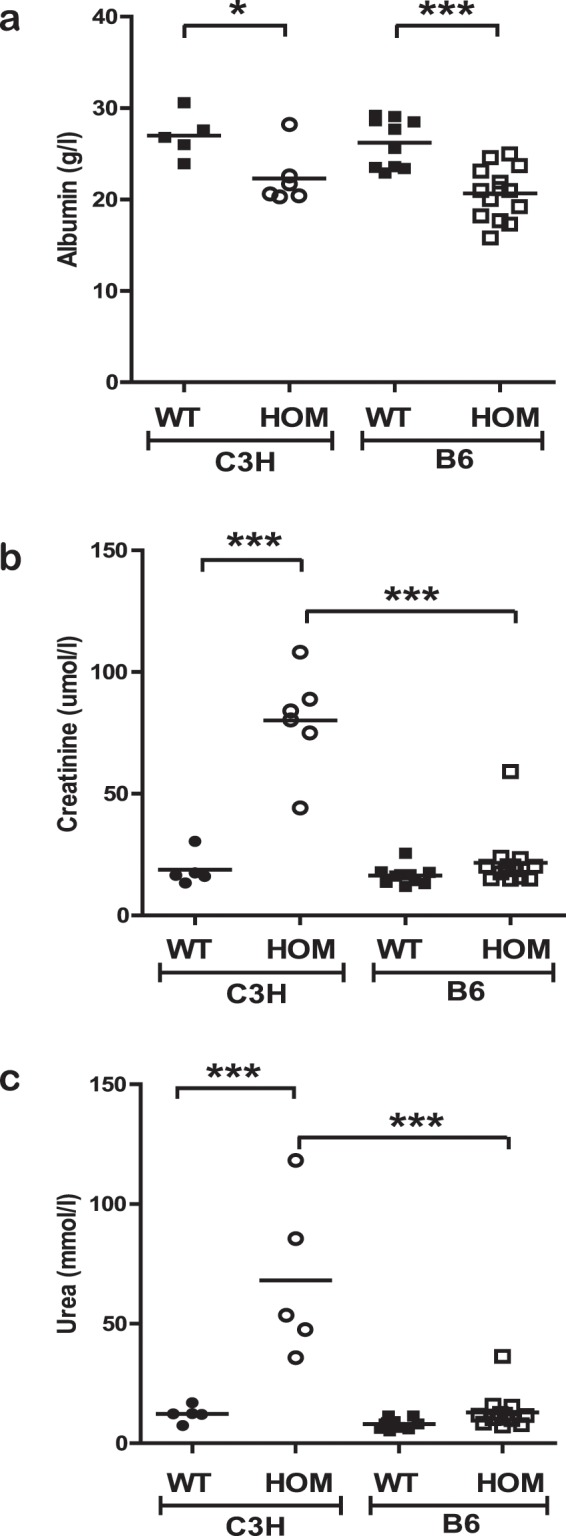
Genetic background influences disease progression in homozygous *Col4a4*^*G400X*^ mice. Terminal plasma clinical chemistry analysis of (**a**) albumin (**b**) creatinine and (**c**) urea reveals that although similar levels of albuminuria are present on both genetic backgrounds there is significant impairment of renal function in C3H-Col4a4^G400X/G400X^ at 7 weeks of age but normal renal function in B6-Col4a4^G400X/G400X^ mice. B6-Col4a4^G400X/G400X^ or C3H-Col4a4^G400X/G400X^ refer to a single outcross of G3 mice to either C57BL/6J or C3H/HeH mice. C3H/HeH wild type n = 5, C3H-Col4a4^G400X/G400X^ n = 6, C57BL/6J wild type n = 9, B6-Col4a4^G400X/G400X^ n = 14. Data shown are mean ± S.E.M. and significance determined using a one-way ANOVA with Tukey’s multiple comparisons test comparing, one-way ANOVA with Tukey’s multiple comparison test *P < 0.05, **P < 0.01, ***P < 0.001.

To further investigate disease pathogenesis on both backgrounds C3H-Col4a4^G400X/G400X^ and B6-Col4a4^G400X/G400X^ mice were terminally bled at 7 weeks of age, when C3H-Col4a4^G400X/G400X^ mice are approaching ESRD, and the levels of several kidney markers were compared. Both strains exhibited similar levels of hypoalbuminemia (Fig. 5a). Serum urea and creatinine levels in C3H-Col4a4^G400X/G400X^ plasma were significantly higher than wild type mice, as expected, whereas the levels of these markers in B6-Col4a4^G400X/G400X^ mice were similar to wild type levels (Fig. 5b,c). Proteinuria, urine output, and water intake showed similar increases on both backgrounds (Figs. S3a–c). Histopathological examination showed that C3H-Col4a4^G400X/G400X^ animals at this time point had severe diffuse glomerulopathy (Fig. 1) whereas B6-Col4a4^G400X/G400X^ exhibited minimal to moderate tubular changes but no significant glomerular changes (Fig. 4c).

**Figure 6:**
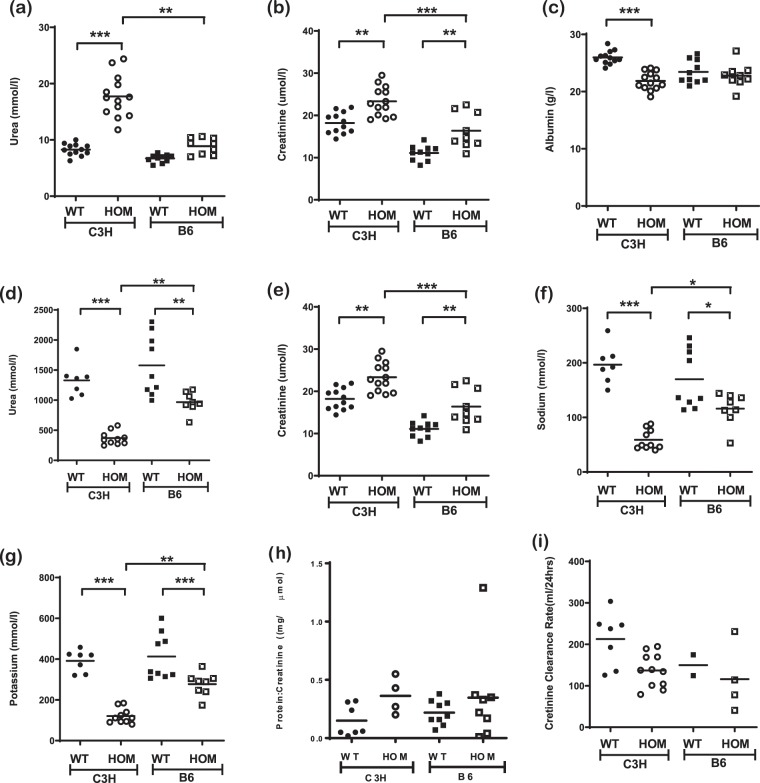
Impaired renal function is detected as early as 4 weeks on both backgrounds. Terminal plasma clinical chemistry analysis of **(a)** creatinine **(b)** urea and **(c)** albumin reveals impairment of renal function in C3H-Col4a4^G400X/G400X^ at 4 weeks of age. C3H-Col4a4^G400X/G400X^ had only mildly elevated creatinine levels at this age. Analysis of 24-hour urine collections revealed disease was detectable on both strains with reduced levels of **(d)** creatinine, **(e)** urea, creatinine clearance rate **(f)** sodium, **(g)** potassium, **(h)** urinary protein:creatinine ratio and **(i)** creatinine clearance on both backgrounds at this age. There were significant differences in all urinary parameters between C3H-Col4a4^G400X/G400X^ and B6-Col4a4^G400X/G400X^ mice indicating a milder disease on the B6 background. B6-Col4a4^G400X/G400X^ or C3H-Col4a4^G400X/G400X^ refer to a single outcross of G3 mice to either C57BL/6J or C3H/HeH mice. C3H/HeH wild type n = 12, C3H-Col4a4^G400X/G400X^ n = 13, C57BL/6J wild type n = 9, B6-Col4a4^G400X/G400X^ n = 9. Data shown are mean ± S.E.M. and significance determined using a one-way ANOVA with Tukey’s multiple comparisons test comparing, one-way ANOVA with Tukey’s multiple comparison test *P < 0.05, **P < 0.01, ***P < 0.001.

The presence of proteinuria does not necessarily result in podocyte damage^18–20^. At 7 weeks of age we observed similar levels of hypoalbuminemia in C3H-Col4a4^G400X/G400X^ and B6-Col4a4^G400X/G400X^ mice, but significantly different levels of serum urea and creatinine, with those in B6-Col4a4^G400X/G400X^ mice being the same as those in wild type mice (Fig. 5). We therefore investigated disease at an earlier time point to determine where the pathogenesis of disease diverged. For the majority of parameters there was a significant difference between wild type and mutant mice (Fig. 6a–g). For serum albumin there was only a difference between wild type and C3H-Col4a4^G400X/G400X^ mice. For the other parameters, both serum and urinary, there was a significant difference between C3H-Col4a4^G400X/G400X^ and B6-Col4a4^G400X/G400X^ mice, with a milder effect of the mutation observed in B6-Col4a4^G400X/G400X^ mice. Although there was not a significant difference in serum albumin levels between C3H-Col4a4^G400X/G400X^ and B6-Col4a4^G400X/G400X^ mice. Whilst there were significant differences detectable at this time in several plasma and urinary parameters, renal function was preserved at this age (Fig. 6h,i) suggesting that, despite significant differences in many parameters in mutant mice, there was a sub-clinical effect on overall renal function at this age. Dipstick analysis revealed significantly higher haematuria, proteinuria and leucocyte levels in C3H-Col4a4^G400X/G400X^ compared to B6-Col4a4^G400X/G400X^ mice (Fig. S4a–c). At this early time point, although evidence of disease was detectable in B6-Col4a4^G400X/G400X^ mice, the overall trend was that disease pathogenesis had already diverged in the two strains by this time point.

**Figure 7:**
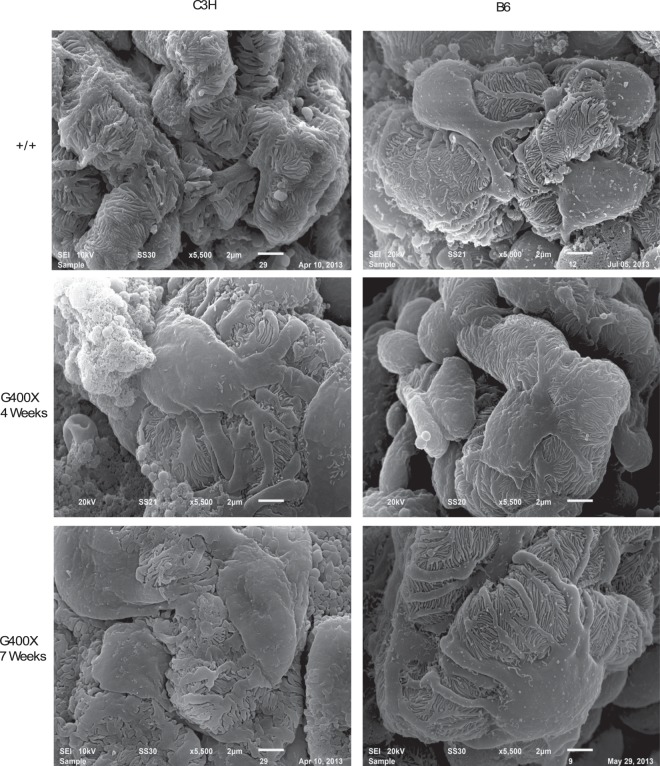
Podocyte morphology is preserved in C3H-Col4a4^G400X/G400X^ mice. Representative scanning electron micrographs of podocytes from C3H-Col4a4^G400X/G400X^ and B6-Col4a4^G400X/G400X^ mice at 4 and 7 weeks of age, with appropriate wild type mice. Foot processes appear flattened and there is reduced spacing between the foot processes. Abnormal podocyte morphology was observed as early as 4 weeks in C3H-Col4a4^G400X/G400X^ mice whereas in B6-Col4a4^G400X/G400X^ mice podocyte morphology was preserved even in the presence of albuminuria at 7 weeks of age. B6-Col4a4^G400X/G400X^ or C3H-Col4a4^G400X/G400X^ refer to a single outcross of G3 mice to either C57BL/6J or C3H/HeH mice.

Glomerular morphology was therefore assessed by SEM. At 4 weeks of age, podocyte cell bodies of C3H-Col4a4^G400X/G400X^ mice appeared larger and swollen, while the foot processes were thickened, flattened, and irregular when compared with Col4a4^G400X/+^ C3H and +/+ control podocytes. By 7 weeks, the podocyte morphology showed more extreme changes in C3H-Col4a4^G400X/G400X^ mice, with podocytes often showing complete effacement. Podocyte morphology in B6-Col4a4^G400X/G400X^ mice showed no significant changes at either time point (Fig. 7).

### Background specific changes in podocyte gene expression

**Figure 8:**
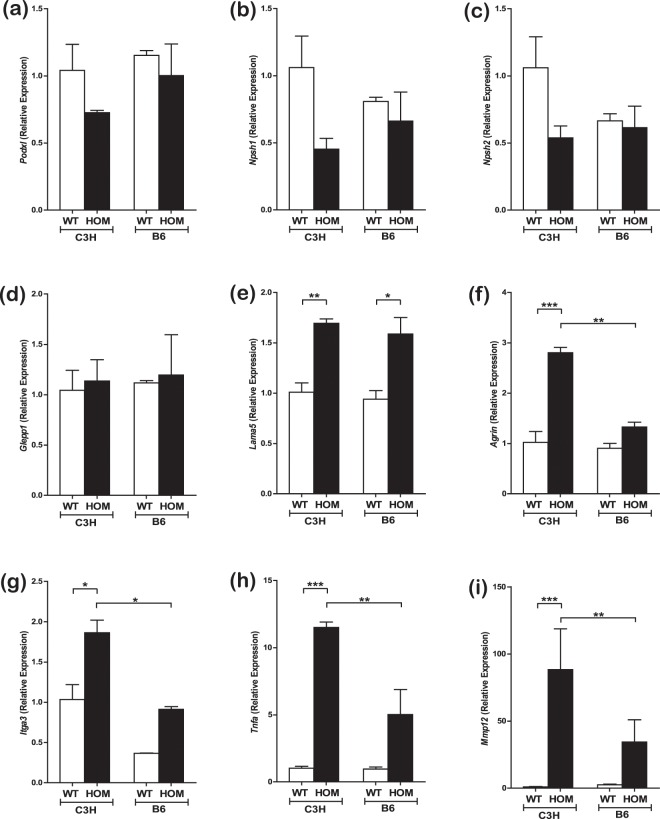
Differences in expression of relevant genes is observed in the two genetic backgrounds. Gene expression levels in C3H-Col4a4^G400X/G400X^ and B6-Col4a4^G400X/G400X^ mice at 7 weeks of age in whole kidney cDNA. **(a)** podocalyxin (*Podxl*) **(b)** nephrin (*Nphs1*) **(c)** podocin (*Nphs2*) **(d)** protein tyrosine phosphatase, receptor type, O (*Ptpro*, GLEPP-1) **(e)** laminin a5 (*lama5*) **(f)** agrin (*Agrn*) **(g)** integrin a3 (*itga3*) **(h)** matrix metalloproteinase 12 (*Mmp12*) and **(i)** tumour necrosis factor (*Tnfa)* expression levels are shown. *Podxl*, *Nphs1* and *Nphs2* showed no changes across strain or genotype whereas *Lama5* increased equally in homozygous mice on both backgrounds. There were significant differences between C3H-Col4a4^G400X/G400X^ and B6-Col4a4^G400X/G400X^ mice in the expression of *Agrn*, *Itga3*, *Tnfa*, and *Mmp12*. B6-Col4a4^G400X/G400X^ or C3H-Col4a4^G400X/G400X^ refer to a single outcross of G3 mice to the either C57BL/6J or C3H/HeH mice. White = wild type (n = 5) Black = homozygous mice (n = 4). Data shown are mean ± S.E.M. and significance determined using a one-way ANOVA with Tukey’s multiple comparisons test comparing, one-way ANOVA with Tukey’s multiple comparison test *P < 0.05, **P < 0.01, ***P < 0.001.

We investigated gene expression of a number of podocyte specific and glomerular related genes in mice from both backgrounds at 7 weeks of age (Fig. 8). There was no difference in *Glepp1, Podxl*, *Npsh1* or *Npsh2* (Fig. 8a–d). However, there was an increase in *Lama5* expression on both backgrounds (Fig. 8e) whereas *Agrin* was upregulated in C3H-Col4a4^G400X/G400X^ mice but not in B6-Col4a4^G400X/G400X^ (Fig. 8f). Similarly, *Itga3* expression increased in C3H-Col4a4^G400X/G400X^ but not in B6-Col4a4^G400X/G400X^ (Fig. 8g). Taken together these data suggest that, whilst similar processes are occurring in these two strains at this time point, there are also differences in the response of podocytes that may influence disease progression.

### Reduced inflammatory markers in B6-Col4a4^G400X/G400X^ mice

A significant difference in the inflammatory response between B6-Col4a4^G400X/G400X^ and C3H-Col4a4^G400X/G400X^ mice at 7 weeks of age was observed. There was reduced expression of matrix metalloproteinase 12 (MMP12) in B6-Col4a4^G400X/G400X^ mice (Fig. 8h), which is expressed by podocytes in response to monocyte chemoattractant protein 1 (MCP-1, *Ccl2*) and has been demonstrated to contribute to disease^31^. TNF-α, another important factor in AS^8^, was reduced in B6-Col4a4^G400X/G400X^ when compared to C3H-Col4a4^G400X/G400X^ mice (Fig. 8i).

**Figure 9:**
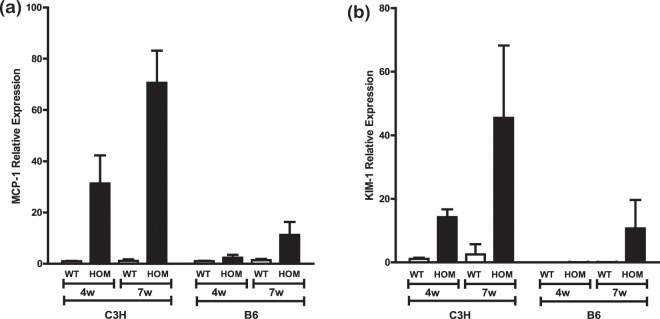
Disease markers slow delayed disease progression in B6-Col4a4^G400X/G400X^ mice. Expression of **(a)** MCP-1 and **(b)** KIM-1 were determined in wild type and homozygous C3H-Col4a4^G400X/G400X^ and C3H-Col4a4^G400X/G400X^ mice at 4 weeks and 7 weeks of age (n = 3). In both cases there was a significant effect of background strain (KIM-1 p = 0.00984, MCP-1 p = 0.00066). B6-Col4a4^G400X/G400X^ or C3H-Col4a4^G400X/G400X^ refer to a single outcross of G3 mice to either C57BL/6J or C3H/HeH mice. An ANOVA was used to compare the linear models fitted to age or age and background strain and assess the probability of a model with background strain providing a better fit.

To further investigate disease progression, we determined the expression of MCP-1 and kidney injury molecule 1 (KIM-1, *Hsvcr1*). MCP-1 is thought to contribute to macrophage recruitment during disease in AS^32^. KIM-1 is expressed primarily by the proximal tubular endothelial cells and its expression increases rapidly upon injury, thus making a potential biomarker of kidney injury (reviewed in^33^). For both MCP-1 and KIM-1 there was a greatly reduced relative level at 4 weeks and 7 weeks of age in B6-Col4a4^G400X/G400X^ mice, with undetectable levels of KIM-1 at 4 weeks of age in these mice (Fig. 9).

## Discussion

Through a phenotype-driven approach we have identified a mouse model of *Col4a4* deficiency with an aggressive disease phenotype. After crossing the mutation onto the C57BL/6J and C3H genetic backgrounds there were significant changes observed between strains, with a divergent pathogenesis from an early time point in these mice that may contribute to the response of the different strains downstream.

To investigate podocyte responses to the abnormal GBM we investigated the expression of relevant genes in homozygous mutant mice and controls. It should be noted that the mice used were at an early back cross 3 generations. Whilst there are similarities between the two strains in some aspects of disease, our studies have identified significant differences reflecting the divergent pathways in disease pathogenesis. The increased expression of *Lama5* has been observed before in models of AS^34^, and was observed on both backgrounds. This has been shown to be associated with an increased permeability of the GBM and hence is thought to contribute to disease pathogenesis^35^. *Itga3* showed increased expression in C3H but not in B6 mice. Integrin α3β1 is an important receptor for laminin 521^36^ and *is* expressed primarily on podocytes. It is important in podocyte foot process formation^37^ and can regulate gene expression^38^. Integrin α3β1 has been demonstrated to be upregulated in another model of AS^39^ and thus the reduced upregulation of *Itga3* in B6-Col4a4^G400X/G400X^ mice, when compared to C3H-Col4a4^G400X/G400X^ mice, could contribute to the lower level of disease in these mice.

There was significant up-regulation of TNF-α on both backgrounds but the expression of TNFα, previously shown to be expressed primarily by podocytes during the development of AS^8^, was significantly lower on the C57BL/6J background. Blockade of this pathway can improve renal function, reduce glomerular damage and improve survival of *Col4a3*^*−/−*^ mice^8^. Therefore, the reduced expression of TNF-α may contribute to the improved prognosis of B6-Col4a4^G400X/G400X^ mice, possibly through reduced podocyte apoptosis^8^. Similarly, there was also greatly reduced expression of *Ccl2*, a chemokine responsible for the recruitment of monocytes, dendritic cells and memory T cells, in B6-Col4a4^G400X/G400X^ mice (Fig. 9).

There was also reduced expression of *Havcr1* (KIM-1) in B6-Col4a4^G400X/G400X^ mice in comparison with C3H-Col4a4^G400X/G400X^ mice, indicating reduced tubular damage^33^, and correlated with a reduced inflammatory response. The slower progression of disease on the C57BL/6J background has allowed us to determine that increased expression of *Havcr1* and *Ccl2* precede changes of serum creatinine, urea, and GFR. Indeed, they can distinguish between disease severity at similar levels of proteinuria (Figs. 5a and 8). Progressive tubular damage has been described in other models of AS and was initially identified as the major determinant of renal failure^21,22^. Modification of the tubular response in a model of AS has been demonstrated to slow disease progression^9^ and the cross talk between the response to the GBM defect and downstream tubular damage may be critical to the pathogenesis of AS. Blockade of KIM-1 itself has also resulted in improved survival via reduced inflammation and tubular necrosis in a model of acute kidney injury^40^. Interestingly, blocking KIM-1 also resulted in reduced recruitment of macrophages. Given that we could detect increases in MCP-1 and KIM-1 expression prior to changes in plasma levels of creatinine and urea, these may prove to be useful urinary biomarkers of disease progression in AS. Indeed, the lower levels of MCP-1 suggest a delayed inflammatory response that is likely to contribute to the slower disease on the C57BL/6J background

The increased survival of B6-Col4a4^G400X/G400X^ mice appears to be due to the persistence of normal podocyte morphology and gene expression. Given the normal GFR of B6-Col4a4^G400X/G400X^ mice at 7 weeks of age, the implication is that the filtration function of the glomerulus is, to a large degree, being maintained. This is in line with previous observations suggesting that C57BL/6J mice with AS may have slower disease progression^6^. Proteinuria has been demonstrated to precede podocyte dysfunction and effacement in a model of Pierson Syndrome, and we have confirmed this in AS^16^. A lack of albumin has also recently been demonstrated to ameliorate disease in an AS model^17^. However, there is also evidence that podocyte morphology can be maintained in the presence of proteinuria^18–20^.

Up regulation of *Col4a6* has been identified as a compensatory mechanism ameliorating disease on the C57BL/6J background^7^. Our breeding strategy means that the X chromosome could not be derived from the C57BL/6J background, thus eliminating the influence of an increased *Col4a6*. A negligible role for COL4A6 in protection against *Col4a3-/-* AS has also recently been demonstrated^41^. Thus, the significant change in disease progression we observed must be due to autosomal loci^6^. Furthermore the C57BL/6J background appears to be protective against the effects of an altered GBM composition as 129 × 1/Sv^7^, FVB^24^, and now C3H strains, all develop more severe disease than C57BL/6J mice in models with GBM abnormalities. Indeed, while we observed a delayed disease on the C57BL/6J background when compared to 129/Sv, previous studies in another Col4a4 mutant line have demonstrated that disease can be further accelerated and was more severe on the DBA/2 background than 129S1/SvImJ^30^. Thus, there may be several pathways that can modify disease severity. Background strain has been shown to influence the composition of the extracellular matrix (ECM)^42^ which correlated with levels of albuminuria. There was increased thickening and splitting of the GBM in FVB mice, which also have fewer glomeruli than C57BL/6J mice, and this may have long term consequences on renal function^43^. A previous study identified significant linkage to two regions, on chromosomes 9 and 16, influencing AS disease progression^6^. We therefore examined published sequence data of the various strains available from the Mouse Genomes Project database^44^ to identify sequence differences that were exclusive to the C57BL/6J strain and not present in 129, C3H/HeH and FVB. We assumed the same gene(s) modify disease on the different backgrounds and focussed on mutations that would affect protein coding.

This preliminary interrogation, whilst not definitive or taking into account the potential effect of copy number variations, revealed some interesting findings. The analysis revealed several relevant polymorphisms in the linkage region on chromosome 9 (Table S2) including: *Adamts7*, associated with inflammatory damage in elderly mice^45^ and *Tpbg*, previously associated with glomerulonephritis^46^. However, the most significant result was a missense polymorphism in myosin 1e (*Myo1e*) resulting in a protein sequence difference at position 73 with a valine residue in C57BL/6J and an isoleucine in the other three strains. *Myo1e* is expressed by podocytes as a component of the slit diaphragm^47,48^; its deletion promotes podocyte injury^49^, and mutations in this gene have been associated with renal disease^50,51^ It has also been identified as a candidate for a modifier of AS disease in a an affected family^52^ carrying a homozygous 118 Lys > Glu mutation. Mutations near the polymorphism in *MYO1E* in C57BL/6J have also been associated with focal segmental glomeruloclerosis^51^ and nephrotic syndrome^50^. An alteration in the function of a protein involved in the regulation of the actin cytoskeleton, and which is a component of the slit diaphragm^48^, would fit with our observations that podocytes in B6-Col4a4^G400X/G400X^ mice undergo slower podocyte effacement. Further investigations will need to be undertaken to confirm or eliminate this polymorphism as a modifier of AS.

In summary, we have confirmed the existence of autosomal modifiers of glomerular basement membrane injury in a novel model of AS, and extended existing studies to demonstrate differences in gene expression and a reduction in inflammatory markers during disease. We have identified a sequence variant in an appropriate genomic region that could contribute to the strain specific variation in disease, but further work is required for conclusive proof that allelic variants of *Myo1e* are a contributing factor. The strain specific differences in responses to an abnormal GBM are helping to deconvolute AS pathogenesis and may point to opportunities for interventions, for example by targeting the response to increased *Lama5* expression. Finally, given the aggressive and early onset of renal failure on the C3H background, the C57BL/6J background may be a better model for trials of therapy as there is a greater window of opportunity for intervention.

## Methods

### Mice

C57BL/6J and C3H-C3pde6b+ inbred mice were maintained in the Mary Lyon Centre in Harwell UK, in specific pathogen-free conditions, with environmental conditions as outlined in the Home Office Code of Practice. Home Office ethical approval was granted under project licence 30/3070 and mice were euthanized by Home Office Schedule 1 methods. Welfare end points included chronic weight loss exceeding 20%, or rapid weight loss of no more than 15%, and excessive urination as well as general indicators of health. All procedures were carried out according to UK Home Office regulations, those laid out in the project license (30/3070), and local ethical guidelines.

### Generation of mutagenized mice

The ENU mutagenesis protocol has been described previously^4^. Briefly, C57BL/6J male mice (G_0_) were treated with ENU doses of 1 × 120 mg kg^−1^, and then 2 × 100 mg kg^−1^with a week between each dose. The mice were then bred with wild-type ‘sighted C3H’ (C3H.Pde6b+) females^26^. The resulting G_1_ males were bred with wild-type C3H.Pde6b + females to produce G_2_ females, which were then backcrossed to their G_1_ fathers to generate G_3_ offspring.

### Linkage analysis and DNA sequencing

DNA from affected mice and littermates was prepared from ear biopsies and used for linkage mapping utilizing the Illumina GoldenGate Mouse Medium Density Linkage Panel (Gen-Probe Life Sciences Ltd, UK). DNA was prepared for whole genome sequencing (WGS) using the Nucleon BACC2 Genomic DNA Extraction System (GE Healthcare Life Sciences), a library generated, and a single lane of paired-end sequencing (100nt) undertaken employing the Illumina HiSeq platform (Oxford Genomics Centre, Wellcome Trust Centre for Human Genetics) and analysed as previously described^25^. The Col4a4 mutation was validated by pyrosequencing on the PSQ HS 96 A pyrosequencer (Biotage AB) using the following primers: Forward TAGGCATGATGGGACCTC, Reverse (biotinylated) GGGCACAGTCGAGTCTTC, Sequencing GGCATGATGGGACCT.

### Metabolic caging

A time course analysis using mice singly housed for 24 hours in metabolic cages (Techniplast) and terminal bleeds were performed on cohorts of homozygous, heterozygous and wildtype mice at 4, 5, 6, 7 weeks. Heterozygous and wildtype mice were also analysed at an additional time point of 20 weeks. Urinary concentrations of creatinine, urea and albumin are from a 24 hour urine collection.

### Clinical biochemistry analysis of plasma and urine

Plasma concentrations of albumin, urea, creatinine, amylase, total protein and glucose and urine concentrations of urea, urinary protein and creatinine were measured on an AU400 Olympus analyser. Lithium heparin blood samples were taken from the retro-orbital sinus under terminal anaesthetic.

### Immunofluorescence analysis

Frozen sections of kidney were stained as described previously^53^. Slides were fixed in 2% paraformaldehyde solution for 10 minutes and x3 washed in PBS before permeabilisation with PBS/1% triton for 15 minutes and then washed again x3. Sections were stained with αCOL4A4 at a 1:100 dilution (clone RH42^53^) and aWT1 (clone C-19, Santa Cruz). After washing, sections were stained with goat anti-rat Alexa Fluor 568 and goat anti-rabbit Alexa Fluor 488 (ThermoFisher) at a dilution of 1:1000.

### Histopathological analysis

Formalin fixed tissues were embedded in paraffin wax and 3um sections were cut using a microtome Finesse ME (Thermo Electron). Kidneys sections were stained with haematoxylin and eosin and analysed by a qualified veterinary pathologist. Pathological findings were scored using a non-linear semi quantitative grading system from 0 to 5 where 0 = no finding in tissue and 5 = whole tissue affected^54^.

### Gene expression analysis

RNA was extracted from whole kidney using the Qiagen RNeasy midi kit and cDNA was synthesised using high capacity cDNA reverse transcription kit (Thermo Fisher Scientific, Waltham, USA). The following TaqMan® (Thermo Fisher Scientific, Waltham, USA) probes The following Taqman probes were employed to quantify expression levels with results being normalised to *Ipo8* expression; *Nphs1*: 00497828-m1, *Itga3*: 0042910_m1, FAT-1: 01200756_m1, MMP12: 0050054_m1, *Posx1*: 00449829_m1, *Pdpn*: 01348912_g1, *Lama5*: 01222029_m1, *TNFa*: 00443260_g1, *Agrin*: 01264853_m1, *Nphs2*: 01292252_m1.

### Electron microscopy

Samples were prepared by fixation by immersion in 2.5% glutaraldehyde in 0.05 M sodium cacodylate buffer pH 7.2 for 2 hours at room temperature, washed twice in 0.05 M cacodylate buffer, then immersed in 1% osmium tetroxide in 0.05 M cacodylate buffer pH 7.2 for one hour at room temperature. Fixed samples were washed 6 times in distilled water, for 3 minutes each dehydrated through an ethanol series at 4 °C: 50% ethanol, 70%, 90% ethanol, for 30 minutes to an hour, depending upon the size of the tissue then immersed in two changes of 100% ethanol for 30 minutes each at 4 °C and transfered to 100% acetone at 4 °C for storage. Critical point drying was carried out using a Leica CPD 300 according to manufacturer’s instructions. After mounting on adhesive carbon discs samples were sputter-coated win a Quorum Q150RS and imaging carried out on a JEOL JSM -6010LV.

### Vision phenotyping

The optokinetic response (OKR) test with the visual-tracking drum has been described elsewhere^55^ with the addition in this study of a video camera to record mouse movements (see www.eumorphia.org/ for protocol). Male and female mice were tested at 7 weeks of age.

### Auditory-evoked brainstem response

Mice were anaesthetized with Ketamine (10%v/v) and Xylasine (5% v/v) administered at the rate of 0.1 ml/10 grams body mass. Animals were placed on a heated mat inside a sound attenuated chamber. Electrodes (Grass Telefactor F-E2–12) were placed subdermally over the vertex (active), right mastoid (reference), and left mastoid (ground). ABR responses were collected, amplified and averaged using TDT System III (Tucker Davies Technology, Alachua, FL, USA) in conjunction with SigCal and SigGen (Biosig) software as previously described^25^. Male and female mice were tested at 7 weeks of age.

## Supplementary information

Supplementary data.
