# Supplementary material for: Modification of an aggressive model of Alport Syndrome reveals early differences in disease pathogenesis due to genetic background
Source: Sci Rep. 2019 Dec 31;9:20398. doi: 10.1038/s41598-019-56837-6 (PMC6938516; doi:10.1038/s41598-019-56837-6)
Supplement: Supplementary file 1 — Supplementary data. [file 41598_2019_56837_MOESM1_ESM.pdf]

Modification of an aggressive model of Alport Syndrome reveals early differences in disease pathogenesis due to genetic background

Sara Falcone, Laura Wisby PhD, Thomas Nicol , Andrew Blease, Becky Starbuck, Andrew Parker, Jeremy Sanderson, Steve DM Brown, Cheryl L Scudamore, Charles D Pusey MD, Frederick WK Tam MD, Paul K Potter

	Mean	SD	n
Urea (Wild type)	8.452	1.427	62
Urea (Mutant)	34.42	8.452	6
Creatinine (Wild type)	11.87	2.370	56
Creatinine (Mutant)	72.67	47.16	6

Table S1. Creatinine and urea levels in wild type and mutant mice at aged 90 days. Mice from the founder pedigree were bled and plasma analysed. Genotypes were subsequently confirmed.

Position (Chr1)	Functional Class	Gene	Ensembl Transcript ID	Transcript SNP Position	Reference sequence	Mutant Transcript Reads	Amino Acid Position	AA Reference	AA Mutant
81501529	Intergenic	.	.	.	.	.	.	.	.
81592544	Intergenic	.	.	.	.	.	.	.	.
81604206	Intergenic	.	.	.	.	.	.	.	.
81730142	Intergenic	.	.	.	.	.	.	.	.
82242238	Intronic	insulin receptor substrate 1	ENSMUST00000069799	.	A	C	.	.	.
82402913	Intronic	rhomboid domain containing 1	ENSMUST00000027322	.	G	A	.	.	.
82515651	Nonsense (Stop)	collagen, type IV, alpha 4	ENSMUST00000087050	1549	G	T	400	G	*
82624993	Intronic	collagen, type IV, alpha 3	ENSMUST00000141994	.	T	C	.	.	.
82630494	Intronic	collagen, type IV, alpha 3	ENSMUST00000141994	.	A	G	.	.	.
82780442	Intergenic	.	.	.	.	.	.	.	.
82784548	Intergenic	.	.	.	.	.	.	.	.
82824427	Intergenic	.	.	.	.	.	.	.	.
82943073	Intergenic	.	.	.	.	.	.	.	.
82997344	Intergenic	.	.	.	.	.	.	.	.
82997500	Intergenic	.	.	.	.	.	.	.	.

Table S2. A list of all point mutations identified in the minimal mapping region. The only mutation affecting the coding sequence was a nonsense mutation in Col4a4. This was confirmed as the causative mutation through subsequent breeding and an absence of COL4A4 protein in mutant animals.

Position (Chr 9)	Gene	dbSNP	C57BL/6 J	129S1 SvImJ	C3H/HeJ	FVB/N J	Functional Class
57254009	1700017B05Rik	rs30485901	A	G	G	G	missense_variant
57256883	1700017B05Rik	rs33628338	A	G	G	G	missense_variant
57257133	1700017B05Rik	rs29739966	T	G	G	G	missense_variant
57258402	1700017B05Rik	rs13465819	T	C	C	C	missense_variant
64038923	1700055C04Rik	rs30355861	A	G	G	G	splice_region_variant
85842983	9330154J02Rik	rs30022245	T	C	C	C	missense_variant
69806902	AC158992.1	rs30323699	C	T	T	T	splice_region_variant
90193461	Adamts7	rs29987966	G	T	T	T	missense_variant upstream_gene_variant
90193821	Adamts7	rs46417067	C	T	T	T	missense_variant upstream_gene_variant
59303491	Adpgk	rs50967282	T	C	C	C	splice_region_variant synonymous_variant
59314743	Adpgk	rs13480222	A	G	G	G	missense_variant
59314812	Adpgk	rs29744020	G	T	T	T	missense_variant
59314908	Adpgk	rs29884714	G	T	T	T	missense_variant
95865570	Atr	rs29782300	C	G	G	G	missense_variant
56241207	C230081A13Rik	rs38848454	A	G	G	G	missense_variant
71724486	Cgnl1	rs33742548	T	C	C	C	missense_variant
97454700	Clstn2	rs29737426	A	C	C	C	missense_variant
97454725	Clstn2	rs30238375	T	G	G	G	missense_variant
62520391	Coro2b	rs33689334	A	G	G	G	missense_variant
87040451	Cyb5r4	rs46143602	A	G	G	G	missense_variant
87042782	Cyb5r4	rs51364509	T	C	C	C	splice_region_variant synonymous_variant
87042881	Cyb5r4	rs45935831	T	C	C	C	splice_region_variant
87055827	Cyb5r4	rs45634827	T	C	C	C	missense_variant upstream_gene_variant
87057232	Cyb5r4	rs29974382	G	A	A	A	missense_variant upstream_gene_variant
87058957	Cyb5r4	rs29588640	G	A	A	A	missense_variant
53919586	Elmod1	rs3690702	A	C	C	C	splice_region_variant
73029705	ENSMUSG00000079469	rs33732168	C	T*	T*	T*	missense_variant splice_region_variant
89856518	ENSMUSG00000098627	rs108309645	G	A	A	A	splice_region_variant
58555164	Gm10657	rs48739782	G	A	A	A	stop_gained upstream_gene_variant
59145774	Gm7589	rs29745656	C	T	T	A	missense_variant
76246391	Hcrtr2	rs29735660	T	C	C	C	missense_variant
76254702	Hcrtr2	rs3685528	A	C	C	C	splice_region_variant synonymous_variant
80316176	Impg1	rs52051539	G	T	T	T	missense_variant
80394203	Impg1	rs46161597	T	A	A	A	missense_variant
80465254	Impg1	rs49899450	C	A	A	A	missense_variant
80465281	Impg1	rs46905549	C	A	A	A	missense_variant
63534380	Iqch	rs214169669	A	G	G	G	splice_region_variant
61918902	Kif23	rs47034601	G	C	C	C	splice_region_variant synonymous_variant upstream_gene_variant
61927046	Kif23	rs29593069	C	T	T	T	missense_variant
77435041	Lrrc1	rs48338099	A	G	G	G	splice_region_variant
86586988	Me1	rs45995457	C	T	T	T	missense_variant
86596203	Me1	rs33750430	A	G	G	G	splice_region_variant
77138432	Mlip	rs48251561	G	A	A	A	missense_variant
77138433	Mlip	rs46656365	A	G	G	G	missense_variant

77164898	Mlip	rs30427253	G	A	A	A	splice_region_variant
72438585	Mns1	rs45900837	T	G	G	G	splice_region_variant
70297336	Myo1e	rs30226946	G	A	A	A	missense_variant
75290928	Myo5c	rs29927685	C	T	T	T	missense_variant
58921760	Neo1	rs30332942	T	C	C	C	splice_region_variant
58656654	Nptn	rs36276964	G	A	A	A	splice_region_variant
88364661	Nt5e	rs29699484	T	C	C	C	splice_region_variant
56126644	Pstpip1	rs40216839	G	A	A	A	missense_variant
98490690	Rbp2	rs33623895	A	G	G	G	splice_region_variant
55975096	Rfpl3s	rs29642778	C	T	T	T	missense_variant
55980794	Rfpl3s	rs46737160	G	C	C	C	splice_region_variant
55980799	Rfpl3s	rs36340363	G	T	T	T	missense_variant splice_region_variant
72617202	Rfx7	rs48028757	A	G	G	G	missense_variant upstream_gene_variant
57446176	Scamp5	rs30192604	A	G	G	G	splice_region_variant
80116600	Senp6	rs29633357	A	C	C	C	missense_variant
80130844	Senp6	rs13461453	G	C	C	C	missense_variant
80143686	Senp6	rs29838871	A	G	G	G	missense_variant
86815426	Snap91	rs3690634	A	G	G	G	splice_region_variant
65475983	Spg21	rs30280565	T	C	C	C	splice_region_variant synonymous_variant
71849844	Tcf12	rs29837273	T	C	C	C	missense_variant
59987366	Thsd4	rs6224703	T	C	C	C	missense_variant
60376053	Thsd4	rs37392865	C	T	T	T	splice_region_variant synonymous_variant
67346504	Tln2	rs38285632	T	C	C	C	missense_variant
85844843	Tpbg	rs4227810	C	A	A	A	missense_variant upstream_gene_variant
57240285	Trcg1	rs51578700	A	T	T	T	splice_region_variant
57242162	Trcg1	rs38051758	C	T	T	T	missense_variant
57242201	Trcg1	rs254741890	A	C	C	C	missense_variant
57244189	Trcg1	rs29880999	G	A	A	A	missense_variant
57245843	Trcg1	rs33771359	T	C	C	C	missense_variant
65851461	Trip4	rs30129889	A	G	G	G	splice_region_variant
65858254	Trip4	rs33753128	A	G	G	G	missense_variant
57929714	Ubl7	rs30066215	T	G	G	G	missense_variant splice_region_variant
67921894	Vps13c	rs3722443	T	C	C	C	missense_variant
88569424	Zfp949	rs29889052	T	A	A	A	missense_variant

Table S3. A list of all variants identified in the linkage region. The Mouse Genomes Project database (http://www.sanger.ac.uk/sanger/Mouse_SnpViewer/rel-1410) was interrogated to identify sequence variants that were exclusive to the protective C57BL/6J strain and are listed here.

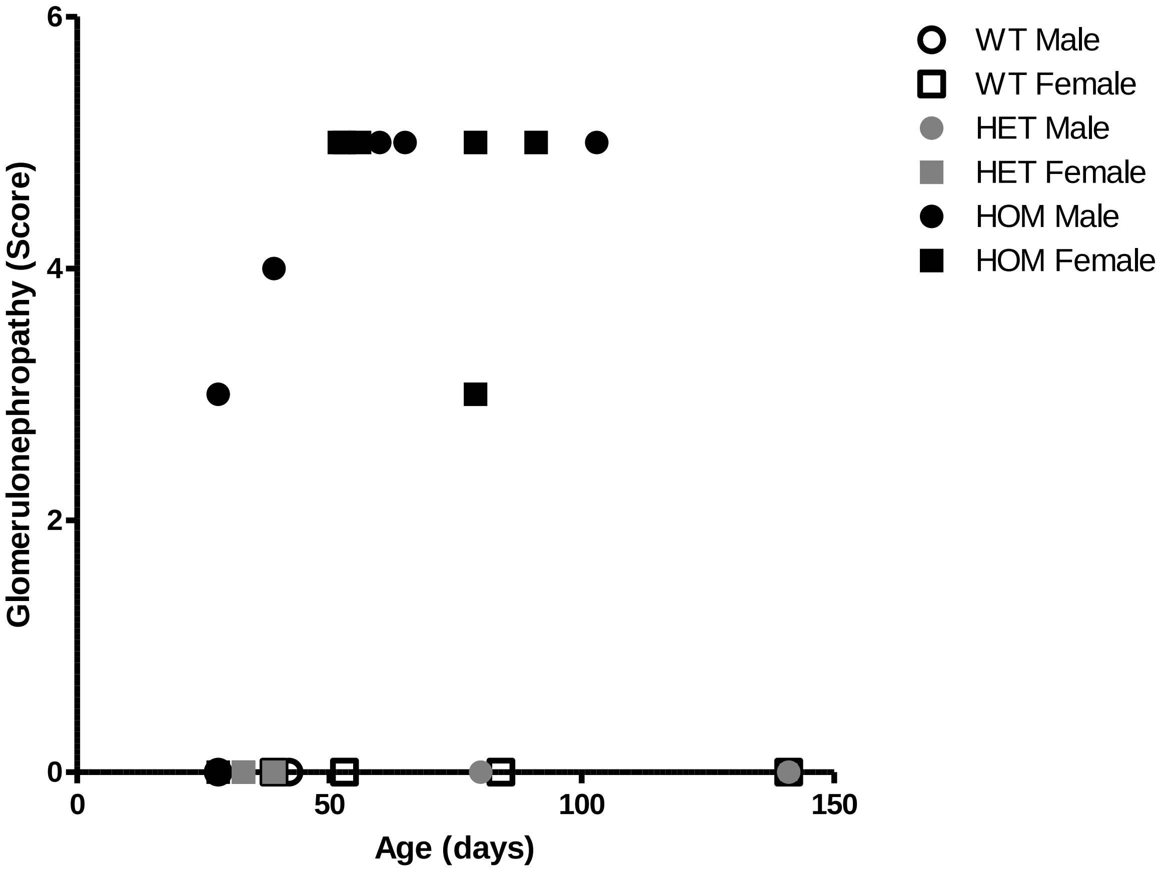

Figure S1. Histopathological findings in C3H-Col4a4$^{G400X/G400X}$ mice. Shown are the glomerulopathy scores against age in days from cohorts of mice aged to 4, 5, 6, and 7 weeks. Glomerulonephropathy was seen in some mice from week 4 and almost all mice were severely affected from week 7. Wild type and heterozygote animals were unaffected.

a

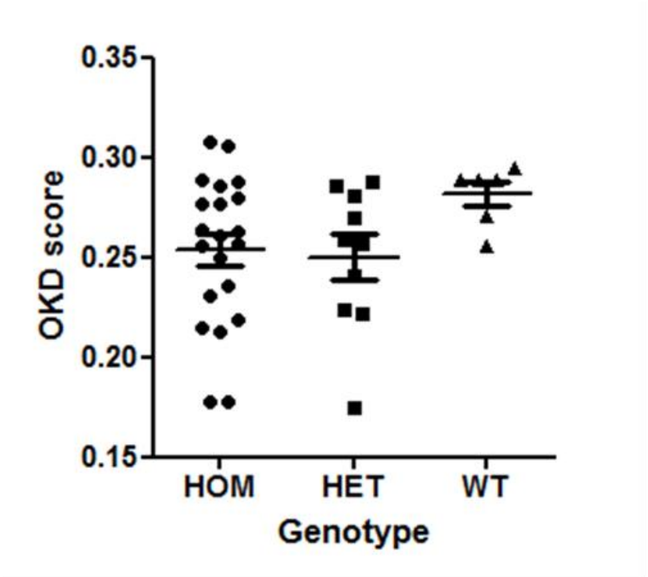

b

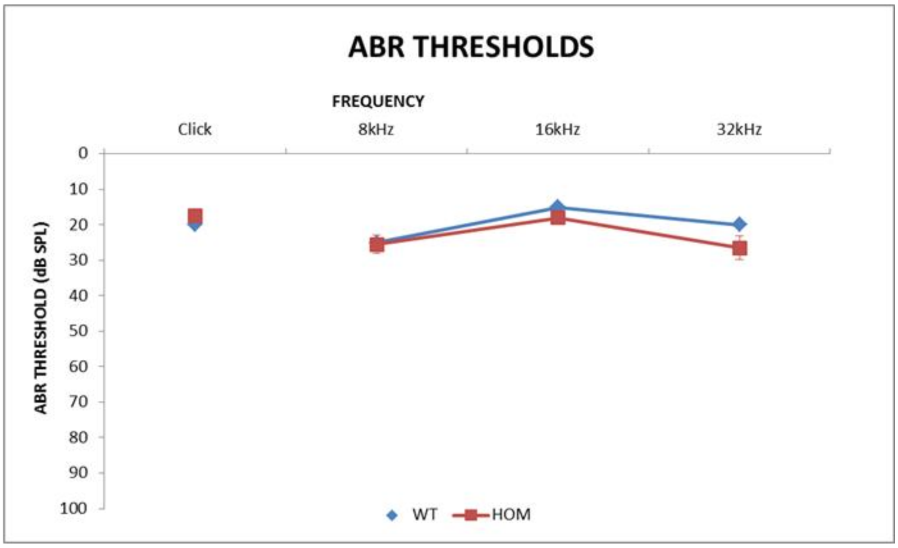

Figure S2. Vision and hearing are unaffected by the Col4a4^{G400X} mutation. Mice were tested for visual acuity **(a)** using the optokinetic drum and hearing assessed by ABR **(b)** at 7 weeks of age in C3H-Col4a4$^{G400X/G400X}$ when disease is prevalent.

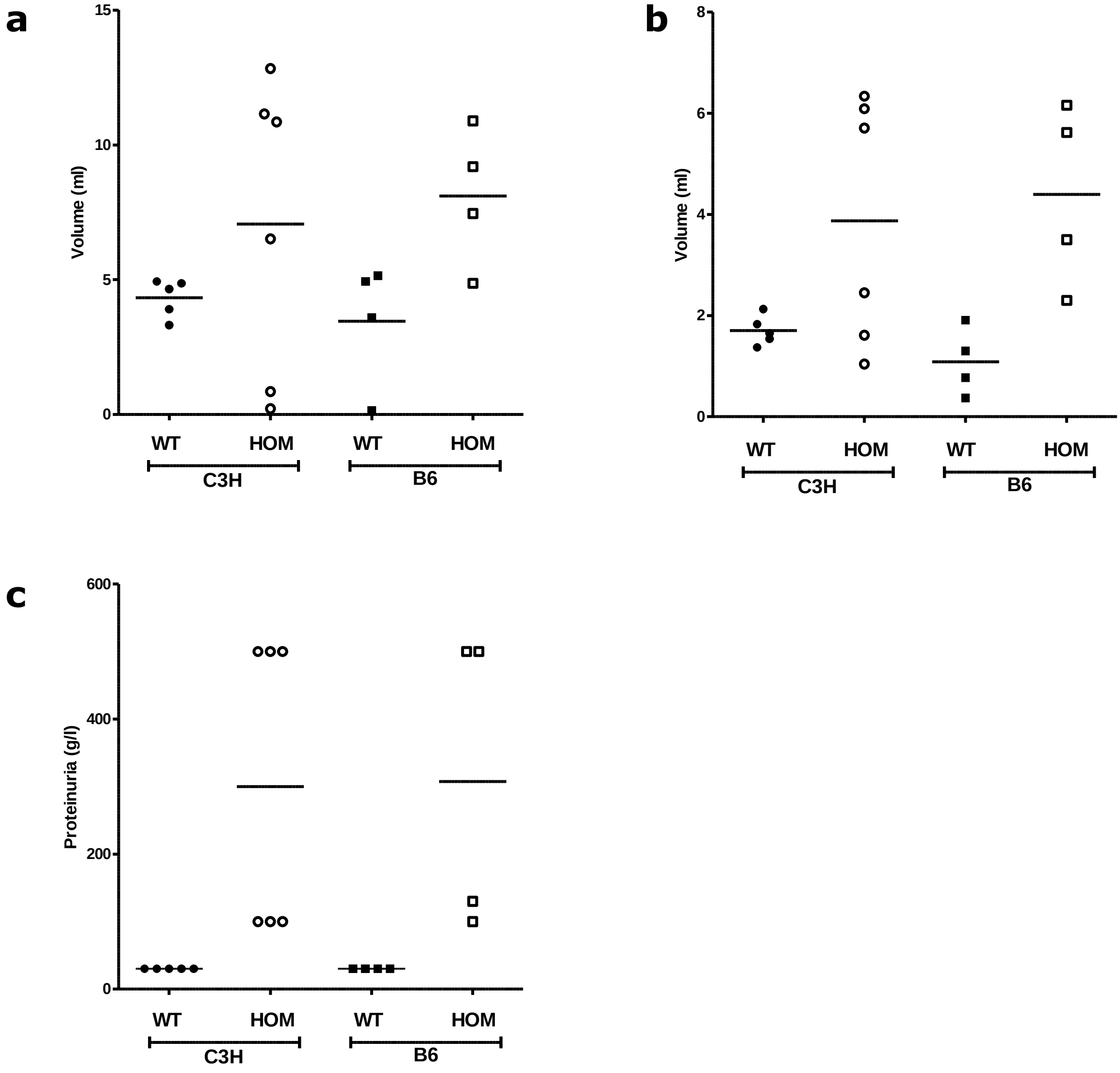

Figure S3. Metabolic cage analysis of C3H-Col4a4$^{G400X/G400X}$ and B6-Col4a4$^{G400X/G400X}$ mice. Metabolic caging data from 7 week old mice showing an increase in **(a)** water intake and **(b)** urine output in homozygous mutant mice on both genetic backgrounds. There was also an increased incidence of proteinuria **(c)** as determined by dipstick in homozygous mutant animals on both backgrounds

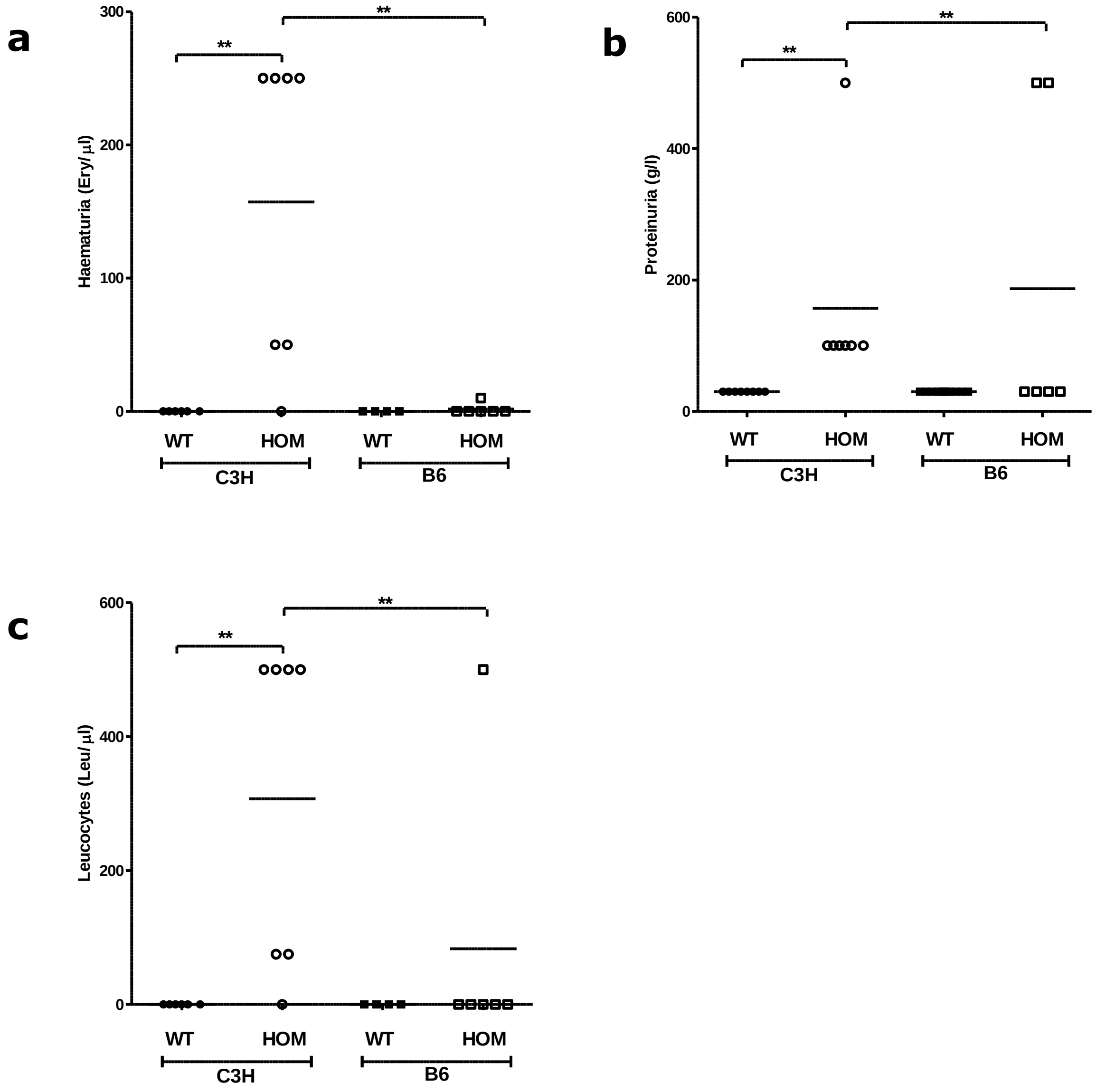

Figure S4. Dipstick analysis of urine from 4 week old mice reveals increased **(a)** haematuria **(b)** proteinuria and **(c)** leucocyte counts in C3H-Col4a4$^{G400X/G400X}$ when compared to B6-Col4a4$^{G400X/G400X}$ mice. Shown is the mean and significance was determined using a one-way ANOVA with Tukey’s multiple comparisons test comparing, one way ANOVA with Tukey’s multiple comparison test * *P*<0.05, ** *P*<0.01, *** *P*<0.001.
